# Greenlip Abalone (*Haliotis laevigata*) Genome and Protein Analysis Provides Insights into Maturation and Spawning

**DOI:** 10.1534/g3.119.400388

**Published:** 2019-08-14

**Authors:** Natasha A. Botwright, Min Zhao, Tianfang Wang, Sean McWilliam, Michelle L. Colgrave, Ondrej Hlinka, Sean Li, Saowaros Suwansa-ard, Sankar Subramanian, Luke McPherson, Harry King, Antonio Reverter, Mathew T. Cook, Annette McGrath, Nicholas G. Elliott, Scott F. Cummins

**Affiliations:** * Aquaculture, CSIRO Agriculture and Food, St Lucia, Queensland, Australia, 4067,; † School of Science and Education, Genecology Research Center, University of the Sunshine Coast, Maroochydore DC, Queensland, Australia, 4558,; ‡ CSIRO Information, Management and Technology, Pullenvale, Queensland, Australia, 4069,; § CSIRO Data61, Acton, Australian Capital Territory, Australia, 2601,; ** Jade Tiger Abalone, Indented Head, Victoria, Australia, 3223,; †† Aquaculture, CSIRO Agriculture and Food, Hobart, Tasmania, Australia, 7000, and; ‡‡ CSIRO Data61, Dutton Park, Queensland, Australia, 4101

**Keywords:** abalone, *Haliotis laevigata*, genome, maturation, spawning

## Abstract

Wild abalone (Family *Haliotidae*) populations have been severely affected by commercial fishing, poaching, anthropogenic pollution, environment and climate changes. These issues have stimulated an increase in aquaculture production; however production growth has been slow due to a lack of genetic knowledge and resources. We have sequenced a draft genome for the commercially important temperate Australian ‘greenlip’ abalone (*Haliotis laevigata*, Donovan 1808) and generated 11 tissue transcriptomes from a female adult abalone. Phylogenetic analysis of the greenlip abalone with reference to the Pacific abalone (*Haliotis discus hannai*) indicates that these abalone species diverged approximately 71 million years ago. This study presents an in-depth analysis into the features of reproductive dysfunction, where we provide the putative biochemical messenger components (neuropeptides) that may regulate reproduction including gonad maturation and spawning. Indeed, we isolate the egg-laying hormone neuropeptide and under trial conditions induce spawning at 80% efficiency. Altogether, we provide a solid platform for further studies aimed at stimulating advances in abalone aquaculture production. The *H. laevigata* genome and resources are made available to the public on the abalone ‘omics website, http://abalonedb.org.

Application of biotechnology to aquaculture is relatively new compared to land-based agricultural species. Shellfish aquaculture represents a large and growing segment of the global aquaculture industry, which includes oysters and abalone from the Phylum Mollusca. To date, the rate of significant advances in mollusc production has been impeded by a lack of genomic resources contributing to the inability to tackle complex traits, such as growth, stress, disease resistance, meat quality, reproduction and environmental adaptation (Elliott 2000). The rapid advancement and application of genomic technologies in research enables a deeper understanding of a species and the means to accelerate production, including closing life cycles and enhancing pathogen sensitivity screening programs compared to predecessors to aquaculture in other innovative land-based agricultural species.

Abalone are commercially valuable molluscs in many countries due to a highly palatable muscular foot. However, depletion of wild abalone stocks has led to significant reductions in commercial catch over the past forty years (Cook 2016). Population structure has influenced this decline, as abalone do not move large distances and are usually found grouped together where they are highly vulnerable to localized overfishing. This has been a major contributor to stock reduction worldwide and the recent collapse of most of the world’s wild fishery has enhanced abalone market potential. Of the 13 tropical and temperate species that occur in Australian waters, the greenlip abalone *Haliotis laevigata* and the blacklip abalone *Haliotis rubra* are of prime commercial importance (Dang *et al.* 2011), contributing $190 million annually (Skirtun *et al.* 2013; Mendoza-Porras *et al.* 2017), which constitutes the second most valuable single edible Australian fisheries export product.

A greater understanding of abalone reproductive biology is critical to the success of closed abalone aquaculture systems. In particular, knowledge of the molecular components required for gonad maturation, such as genes and proteins is needed, which may be intimately associated with the neuroendocrine system. Asynchronous gonad maturation leads to spawning inefficiency and is a major limiting factor impeding progress toward improved complex traits through selective breeding (Elliott 2000; Hayes *et al.* 2007). Spawning is a complex biological process affected by various physiological systems (*e.g.*, endocrine, muscular, sensory) and external stimuli. For example, temperature, atmospheric pressure (*i.e.*, thunderstorms), water quality and anthropogenic pollutants (Jobling *et al.* 2004; Mendoza-Porras *et al.* 2017). Current methods to artificially induce abalone spawning in aquaculture makes use of minor stressors, such as hydrogen peroxide or ultra-violet irradiation (or ozone) (Kikuchi and Uki 1974). However, this spawning induction approach is inefficient, and it therefore requires many generations to make substantive genetic gains in temperate abalone (Elliott 2000; Hayes *et al.* 2007).

In this study, we sequenced the *H. laevigata* genome with a focus on extending our understanding of reproduction and maturation in abalone. Multi-species sequence phylogeny and subsequent identification of molluscan-type neuropeptide genes validate this resource. High-throughput discovery mass spectrometry reveals several central nervous system (CNS) neuropeptides that change in abundance during gonad maturation. And finally, we validate our hypothesis that one neuropeotide, the egg laying hormone (ELH) can stimulate spawning in *H. laevigata*.

## Methods & Materials

### Genome sample collection and nucleic acid preparation

An individual cultivated *H. laevigata* abalone approximately 3 years old was provided by a commercial abalone farm in South Australia, Australia, for sequencing the genome from foot muscle tissue. The individual was a female to reduce heterogeneity of the sample. Organs collected from the same individual including CNS, gonads, muscle, radula, stomach, hepatopancreas, buccal mass, mantle, cephalic tentacles, epipodial tentacles, and gills were frozen at -80° for transcriptome sequencing to provide *de novo* evidence to annotate the genome. For the genome assembly, DNA was extracted from 100 mg of abalone foot muscle using the CTAB method (Doyle and Doyle 1987). In brief, 1 mL of CTAB (2% hexadecyltrimethylammonium bromide, 100 mM TrisHCl, 20 mM EDTA, 1.4 M sodium chloride, 0.2% *β*-mercaptoethanol, 0.1 mg/mL proteinase K) was added to the tissue sample and incubated at 55° for 16 h. RNA was removed by adding 2 *μ*L of RNAse A (10 mg/mL) and incubating at 37° for 1 h. DNA was purified by adding a 1:1 (v/v) of chloroform:isoamyl alcohol (24:1) and mixing by inversion followed by room temperature centrifugation at 14,000 x *g* for 25 min. The aqueous phase was transferred to a new tube and a 1:1 (v/v) of phenol:chloroform:isoamyl alcohol (25:24:1) added before mixing by inversion and centrifugation at 14,000 x *g* for 20 min at room temperature. The aqueous phase was transferred to a new tube, and a 1:1 (v/v) of chloroform:isoamyl alcohol (24:1) was added, before mixing by inversion and centrifugation at 14,000 x *g* for 15 min at room temperature. The aqueous phase was transferred to a fresh tube and DNA precipitated overnight at -20° by addition of cold 100% isopropanol. The DNA pellet was recovered by centrifugation at 14,000 x *g* for 25 min. The DNA was washed twice, first with 70% ethanol followed by 100% ethanol by centrifugation at 14,000 x *g* for 15 and 10 min, respectively. The DNA was air dried and re-suspended in Tris-EDTA. DNA quality was assessed by visualization on a 1.2% agarose gel and quantified using a NanoDrop spectrophotometer (Thermo Scientific, Waltham, MA, USA). Whole transcriptome sequencing of the CNS, gonads, muscle, radula, stomach, hepatopancreas, buccal mass, mantle, cephalic tentacles, epipodial tentacles, and gills was undertaken to inform the *de novo* abalone genome assembly process, and assist with genome annotation. Total RNA was extracted using the Trizol (Invitrogen, Waltham, MA USA) method, as per the manufacturer’s instructions. RNA quality was assessed using a Bioanalyzer 2100 (Agilent Technologies, Santa Clara, CA).

### Sequencing and assembly

DNA was subject to the TruSeq Nano DNA Library Prep construction (Illumina, SanDiego, CA, USA) for 100 bp and 345 bp paired end sequencing and Nextera Mate Pair Library Prep construction (Illumina) for 4 Kb, 7.5 Kb, 10 Kb mate pair sequencing using the Illumina 2500 platform Australian Genome Research facility (AGRF) (Melbourne, Australia). Total RNA was subject to the TruSeq Stranded mRNA library prep construction (Illumina) for 100 bp paired-end sequencing for the transcriptome using the Illumina HiSeq 2500 platform, AGRF (Melbourne, Australia). Illumina raw read quality was assessed using FastQC (Andrews 2010) (Babraham Bioinformatics) before and after adaptor removal, and trimming of raw reads with Trimmomatic v0.32 (Bolger *et al.* 2014) (TruSeq3-PE adapters, leading 5 bp, trailing 5 bp, sliding window 4:15, and retaining reads with a minimum size of 50 bp) to retain 99% of reads for assembly. The final draft genome was assembled with ABySS v 1.5.2 (Simpson *et al.* 2009), k-mer size of 63, and a minimum of 10 pairs to build a contig, followed by an additional scaffolding step with SSPACE (Boetzer *et al.* 2011) using the 4 Kb, 7.5 Kb and 10 Kb mate pair reads. The final assembly was chosen based on the following performance measures: genome size (with/without N), the number of scaffolds (> 2kb), mean scaffold length, longest sequence, N50 and NG50. Quantitative assessment to measure genome completeness was undertaken using BUSCO which assesses the genome against a set of evolutionarily-informed expectations of gene content from 978 near-universal single-copy orthologs selected from OrthoDB v9 (Simão *et al.* 2015) (Table 1). To assist with evidence-based annotation of the genome, a *de novo* transcriptome assembly was performed using Trinity v 2.2.0 (Grabherr *et al.* 2011; Haas *et al.* 2013). SRA data are available from NCBI BioProject PRJNA433241 (https://www.ncbi.nlm.nih.gov/sra/).

**Table 1:** Summary of *Haliotis laevigata* genome assembly statistics

GENOME ASSEMBLY STATISTICS	
Expected genome size	1.54 Gb
Total length (scaffolds)	1.71 Gb
Number of scaffolds (>2kb)	63,588 bp
Mean scaffolds	26,815 bp
Longest sequence	1,091,339 bp
N50 length (scaffolds)	86,085 bp
G+C content	40%

BUSCO GENOME COMPLETENESS STATISTICS	
Complete Single-Copy BUSCOs	827
Complete Duplicated BUSCOs	22
Fragmented BUSCOs	86
Missing BUSCOs	45
Total BUSCO groups searched	978

### Genome annotation

Genome annotation was completed with the Maker v2.13.8 (Cantarel *et al.* 2008) annotation pipeline which includes the *ab initio* predictors SNAP (Korf 2004), and Augustus v 3.1.0 (Stanke and Waack 2003; Stanke *et al.* 2006a,b). Annotation used evidence retrieved from the *de novo H. laevigata* assembled whole transcriptome, NCBI *Haliotis spp*. expressed sequence tags and mRNA, Uniprot *Haliotis spp*. proteins, and the *Crassostrea gigas* reference proteome (Zhang *et al.* 2012) *ab initio* gene predictors were trained on the post-evidence based annotation data. Functional annotation was completed with BLAST2GO (Conesa *et al.* 2005) (Figure S1), and the annotated genome and transcripts were imported into GBROWSE (Stein *et al.* 2002) and Sequence Server (Priyam *et al.* 2015) available on the AbalOmics Community website (http://abalonedb.org). The genome has been deposited at DDBJ/ENA/GenBank, under the accession VKKT00000000. The resulting annotated predicted proteins and transcriptome data were subsequently used in mass spectroscopy protein databases to interpret maturation data.

### Phylogenetic analysis for genome assembly validation

Protein sequence data for a diverse set of 13 phyla consisting of molluscs, brachiopods and annelids, including *Pinctada fucata* (Takeuchi *et al.* 2012), *Bathymodiolus platifrons* (Sun *et al.* 2017), *Modiolus philippinarum* (Sun *et al.* 2017), *Patinopecten yessoensis* (Wang *et al.* 2017), and *Haliotis discus hannai* (Nam *et al.* 2017) were downloaded as directed by the corresponding publications. The *in-silico* proteomes of *Crassostrea gigas*, *Lottia gigantea*, *Octopus bimaculoides*, *Lingula anatina*, *Capitella teleta*, and *Helobdella robusta* were obtained from the ENSEMBL database (https://www.ensembl.org/). The amino acid sequences of *Aplysia californica* were downloaded from the GENBANK data resource (https://www.ncbi.nlm.nih.gov/genbank/). Only sequences containing greater than 50 amino acids were included in the analysis. A reciprocal BLAST-hit approach was performed using the complete proteomes to obtain orthologous proteins from different species. As suggested by Duret *et al.* (1994), the threshold for a significance match was set based on the sequence length (L) and similarity score (S in BLASTp program) as: S = 150 for L < 170 amino acids, S = L-20 for L < 170 amino acids and S = 35 for L < 55 amino acids. These thresholds are low enough to detect all homologous sequences that diverged since vertebrate radiation and are high enough to avoid excessive noise due to the presence of segments of low compositional complexity shared by many gene families. This process produced 748 orthologous genes that were present in the thirteen genomes listed previously.

Amino acid sequences for 748 genes were concatenated to create a super gene. The concatenated sequences from thirteen species were aligned using *MUSCLE* (Edgar 2004) by selecting default settings. After removing alignment gaps from all sequences 127,598 amino acids were available for further analysis. This multiple sequence alignment was used to infer the phylogenetic relationship between the species and the maximum likelihood based *RaxML* (Stamatakis 2014) was used for this purpose. A gamma distribution was used to model the rate variation among sites and four rate categories were chosen. To model substitutions between amino acids we opted for the LG (Le and Gascuel) substitution matrix (Le and Gascuel 2008) and used empirical amino acid frequencies. The species *Helobdella robusta* and *Capitella teleta* were set as outgroups. A bootstrap resampling procedure with 100 pseudo-replicates was used to obtain statistical confidence for each bifurcation (node) of the phylogenetic tree. The software *Figtree* (http://tree.bio.ed.ac.uk/software/figtree/) was used to view and print the tree generated by RaxML.

In order to estimate the divergence times between molluscan species a Bayesian statistics based *MCMCtree*(dos Reis and Yang 2011) method was employed. The amino acid sequence alignment was used for this analysis and the maximum likelihood tree obtained from the *RaxML* program was used as the guide tree. The following fossil ages were used to calibrate the tree: 531-581 million years (MY) for the spilt between Annelida - Mollusca (Spiralia) (Benton *et al.* 2009; Simakov *et al.* 2015), a minimum age of 330 MY for Pectinoida–Ostreoida (Pteriomorpha) (Mergl *et al.* 2001), a minimum age of 260 MY for Crassostrea–Pinctada (Ostreoida) (Nakazawa and Newell 1968), and 500-550 MY for Gastropoda–Bivalvia (Pleistomollusca) split (Erwin *et al.* 2011). We also fixed a maximum age of 600 MY for the root of the tree (root age). To obtain the Hessian matrix for the protein data, the *codeml* program of the software PAML (Yang 2007) was used. Using the WAG+Gamma (Whelan and Goldman 2001) model of amino acid substitution matrix and the four calibration times listed above the divergence times were estimated. The results of *MCMCtree* were checked for convergence and the time-tree generated by this program was viewed using *Figtree*.

### Tissue-specific gene expression

The relative tissue-specific expression of genes can provide information on the function of a gene. A genome guided transcriptome assembly was completed against the *H. laevigata* genome with reads mapped using TopHat v 2.1.0 (Trapnell *et al.* 2009, 2012, Kim and Salzberg 2011; Kim *et al.* 2013). This was followed by quantification of each gene in each tissue using the Cufflinks suite (Trapnell *et al.* 2012) of tools including Cufflinks, Cuffmerge, Cuffquant and Cuffnorm. The quantified tissue-specific expression data were converted to Z-score to explore the tissue-specific expression of genes in various tissues and identify whether genes are over-expressed or under-expressed. A Z-score for a tissue sample refers to the standard deviation away from the mean of expression in all tissue samples, for the formula as shown below where *x* represents the expression in the tissue sample; *μ* represents the mean expression in all the tissue samples, and *δ* represents the standard deviation of expression in all the samples:

Z=(x−μ)/δ (1)

We then used the generated Z-scores for comparison of gene patterns in different tissue types including the ganglia (CNS), gonads, muscle, radula, stomach, hepatopancreas, buccal mass, mantle, cephalic tentacles, epipodial tentacles, and gills. The Z-score threshold values -2 and 2 were used to determine tissue-specific gene expression profiles for each gene, which corresponds to the absolute statistic P-value <0.05. This data was used to explore gene localization patterns (*e.g.*, gonad, ganglia, muscle etc.) in tissues at a single point in time in a single female adult abalone. This data was not intended for differential gene expression analysis which would require replication of transcriptome data from different animals at the same stage of development at the same point in time.

### Abalone neuropeptide precursor prediction and comparative analysis

Neuropeptides are secreted out of the cell, which is facilitated by signal peptides in the precursor protein form. To systematically identify putative neuropeptides in the CNS of *H. laevigata*, we utilized the default settings of five bioinformatics tools on all putative CNS proteins derived from the annotated genome to predict the presence of a signal peptide [SignalP 3.0 (Bendtsen *et al.* 2004) and PrediSi (Hiller *et al.* 2004)], and any transmembrane domains, or signaling domains (TMHMM 2.0 (Krogh *et al.* 2001), SMART (Schultz *et al.* 1998; Letunic *et al.* 2015) and HMMTOP 2.1 (Tusnády and Simon 2001). The resultant proteins were used as potential neuropeptide input to NeuroPred (Hummon *et al.* 2003) to predict cleavage products. Schematic diagrams of protein domain structures were prepared using Domain Graph (DOG, v 2.0) (Ren *et al.* 2009). For protein sequence alignments, MEGA 5.1 (Tamura *et al.* 2011) was used with the clustalW protocol and utilizing the Gonnet protein weight matrix. A representation of NPPs in different species of mollusc was prepared using Cytoscape software (Shannon *et al.* 2003).

### Neuropeptide analysis during abalone reproductive maturation

#### Experimental design and statistical rationale:

Gonad maturation was compared with age every two months for one year beginning in autumn by randomly collecting 25 males and 25 females of each sex. The number of samples at each sample time point was determined by the requirement to observe all stages of maturation [*e.g.* visual gonad index (VGI) stages 0-3] at each time point in a representative sample from the same cohort, while considering the destructive nature of sampling at two commercial abalone farming operations in South Australia and Tasmania. Phenotypic measures collected included water temperature, sex, weight, and VGI (Kikuchi and Uki 1974) (Table 2). The difference in size over the summer months (December to February) is attributed to harvesting of the larger abalone in the older commercial population prior to collection of these samples.

**Table 2:** Seasonal and phenotypic characteristics of samples collected for neuropeptide analysis during *Haliotis laevigata* reproduction

Month	Season	Temperature (°) *^a^*	Age (months)	Mean weight (g)	Mean length (cm)	Visual Gonad Index (M *^b^* / F *^c^*)
April	Autumn	16.0	15	37.50	64.4	0.0 / 0.0
	Autumn	17.0	42	35.28	66.5	0.0 / 0.0
June	Winter	15.2	17	43.70	69.3	0.8 / 0.4
	Winter	13.0	44	42.49	67.7	0.7 / 0.6
August	Winter	14.4	19	40.40	67.3	1.1 / 1.0
	Winter	10.9	46	—	—	— / —
October	Spring	16.7	21	38.94	65.5	1.6 / 1.2
	Spring	14.0	48	45.32	69.0	1.6 / 1.4
December	Summer	18.7	23	50.52	71.2	2.3 / 2.2
	Summer	18.0	50	37.57	65.3	2.2 / 2.4
February	Summer	20.1	25	70.79	78.3	1.7 / 0.4
	Summer	20.0	52	46.09	68.6	1.9 / 1.6

a Water temperature.

b Male.

c Female.

A minimum of three biological replicates for each sex and consistent VGI were extracted individually. The entire CNS was used for each sample preparation for neuropeptidome analysis, therefore, there were no technical replicates in this study. This is acceptable as samples were selected based on a consistent VGI at the same time-point in the maturation cycle (VGI 0, 1 and 2) and season (autumn, spring and summer, respectively) of the year. Controls were samples at VGI of 0 where there is no evidence of gonad development. Samples were initially used for discovery peptidomics and then re-analyzed through a comparison of peak areas to assess relative neuropeptide levels during maturation. Statistical analysis using ANOVA was selected to test the statistical significance of the main effect of two discrete experimental design variables, in our case VGI and sex, and their interaction in abalone at 1 year and 3 years.

#### Experiment:

*H. laevigata* samples were collected from individuals sourced on-site at two Australian commercial abalone farms located in South Australia and Tasmania. The first group consisted of juvenile’s ca. 15 months entering their first year of sexual maturation (1^st^), while the animals in the second group were entering their third year of sexual maturation (3^rd^) at the start of the sampling period. Overall, the 3^rd^ year group was stunted in size compared to the 1^st^ year group for their age. Abalone were euthanised with 360 mM magnesium chloride prior to dissection of CNS. Samples were stored in cryogenic vials, snap frozen in liquid nitrogen, and stored at -80° for laboratory processing. The original CNS transcriptome from adult abalone may not have provided a complete representation of all abalone neuropeptide precursors (NPPs), therefore, to expand the NPP database, additional sequencing was undertaken on two pooled (n = 3) groups of ganglia. The first of these was juvenile’s (pre-gonad development) and the second was from mature adults (VGI 2-3) (Kikuchi and Uki 1974). Total RNA was subject to the TruSeq Stranded mRNA library prep construction (Illumina) for 100 bp paired-end sequencing for the transcriptome using the Illumina HiSeq 2000 platform, BGI Tech Solutions (Hong Kong). SRA data are available from NCBI BioProject PRJNA433241 (https://www.ncbi.nlm.nih.gov/sra/).

To extract peptides from the samples, CNS were thermally treated to denature proteolytic enzymes, and then peptides were extracted using acetic acid as previously described for mice (Svensson *et al.* 2003) and cattle (Colgrave *et al.* 2011) hypothalamus tissue. In brief, 100 mg of CNS tissue was mixed with 20 *μ*L of 0.5% acetic acid and then centrifuged at 14,000 x *g* for 30 min at 4°. Peptides smaller than 10 kDa were isolated by filtration using Microcon YM-10 filter devices (Millipore) and centrifuged at 20,000 x *g* for 90 min at 4°. Samples were vacuum dried and stored at -20° until analysis. Peptides were chromatographically separated using a Shimadzu Prominence LC20 HPLC system with a ZORBAX 300SB-C18 column (75 *μ*m × 15 cm, 3.5 *μ*m) (Agilent Technologies). Samples were reconstituted in 1% formic acid at 2 *μ*L/mg of tissue and then diluted 1:5 for an injection volume of 20 *μ*L. A linear gradient at a flow rate of 300 nL/min from 2–40% solvent B over 44 min was utilized where solvent A was 0.1% formic acid and solvent B was 0.1% formic acid in 90% acetonitrile. Eluent was directed into the nanoelectrospray ionization source of the TripleTOF 5600 system (SCIEX, Redwood City, USA). Data were acquired in two modes. In the first mode, peptide identification was performed using an information-dependent acquisition (IDA) method and in the second only MS analysis was performed for the purpose of peptide quantification. The IDA method consisted of a high-resolution TOF-MS survey scan followed by 20 dependent MS/MS scans. All MS analyses were performed in positive ion mode over the mass range m/z 300 – 1800 with a 0.5 s accumulation time. The ion spray voltage was set to 2400 V, the curtain gas was set to 25, the nebulizer gas to 12 and the heated interface was set to 180°. In IDA mode, MS/MS spectra were analyzed over the range m/z 300 - 1800 using rolling collision energy for optimum peptide fragmentation. Precursor ion masses were excluded for 8 s after two occurrences. The data were acquired and processed using Analyst TF 1.5.1 software (SCIEX). Data are available via ProteomeXchange with identifier PXD009218 (http://www.proteomexchange.org/).

Peptides were identified by searching against the NPP database (44 sequences; Figure S2) using ProteinPilotTM 4.5 software (SCIEX) with the Paragon algorithm (Shilov *et al.* 2007). Search parameters were defined as no cysteine alkylation and no digestion enzyme. The ID focus was on biological modifications and thorough identifications. The resultant peptide identifications were imported into PeakViewTM 1.1.0.0 software (SCIEX). The peak areas for all peptides with confidence greater than 80% were exported to MarkerViewTM 1.2.1.1 software (SCIEX) for statistical analyses of reproduction related candidates. The abundance of each peptide was compared using an analysis of variance (ANOVA) model that contained the main effects of sex and VGI and their interaction. The estimates of least-square means for the sex by VGI interaction were used to identify peptides with statistically different abundance between the two sexes at either one of the three VGI levels. Statistical analyses were performed using the Procedure GLM of SAS 9.4 (SAS Institute Inc., Cary, NC, USA). The least-square estimates were further scrutinised in the form of a heatmap generated using the PermutMatrix software (Caraux and Pinloche 2005). The average abundance for each peptide was converted to a Z-score to explore the overall trend in abundance of neuropeptides involved in maturation between abalone entering their 1^st^ year of reproduction and 3^rd^ year of reproduction.

### Egg laying hormone (ELH) spawning assay

Spawning assays with synthetic C-terminal amidated ELH (GLSINGALSSLADMLSAEGQRRDHAAALRLRQRLVA-NH) were undertaken at a commercial abalone hatchery in Victoria, Australia. Destructive sampling of highly valuable elite broodstock and the limited number of individual aquaria for the trial restricted biological replicates (n = 10) for each treatment group. An equal number of adult male and female *H. laevigata* of a similar size and age (2-3 years old) were conditioned separately at 16° for approximately four months. Animals were selected with similar gonad condition (VGI 2) and randomly allocated to three treatment groups for males and females consisting of five animals each. Abalone were placed into individual aerated spawning tanks in a controlled temperature environment room to maintain water temperature at 15° for the trial. The treatment groups received either a 100 µl injection of molluscan saline (HEPES 13 g, NaCl 25.66 g, KCl 0.82 g, CaCl_2_ 1.69 g, MgCl_2_ 10.17 g, Na_2_SO_4_ 2.56 g, dH_2_O to 1 L, at pH 7.2) (Chansela *et al.* 2008) (control group), or 1.0 µg/g body weight (without shell) ELH in molluscan saline. Abalone were observed for any changes at 2 h post-injection and then the following day for spawning as demonstrated by the presence of gametes in the tanks. All individuals were euthanised (injection of 250 µl 360 mM MgCl_2_) and the VGI was assessed after dissection to confirm spawning based on gonad condition.

### Data availability

This Whole Genome Shotgun project has been deposited at DDBJ/ENA/GenBank under the accession VKKT00000000. The version described in this paper is version VKKT01000000. The raw Illumina reads of the *Haliotis laevigata* draft genome and transcriptomes are deposited in the NCBI GenBank as Sequence Read Archive (SRA) under the following BioProject PRJNA433241. Mass spectrometry data are available via ProteomeXchange with identifier PXD009218 (http://www.proteomexchange.org/). The *Haliotis laevigata* draft genome and associated resources including GBROWSE and Sequence Server are available at the abalone community portal, http://abalonedb.org, developed as part of this project. The website is available for contributions by the abalone community. Supplemental data are available on figshare for the following figures and data files: Figure S1. Summary of annotation statistics for the *Haliotis laevigata* genome. (Figure_S1_ Genome_annotation_statistics.pdf). File S1. Gene ontology of the *ab-initio* predicted genes from the *Haliotis laevigata* genome. [File_S1_Transcript_annotation.xlsx]. File S2. Summary of identified genes encoding putative full-length or partial-length neuropeptide precursors from the *Haliotis laevigata* database. Homolog neuropeptide precursors are also shown for *Theba pisana*, *Aplysia californica*, *Lottia gigantea*, *Charonia tritonis*, *Biomphalaria glabrata*, *Crassostrea gigas*, *Saccostrea glomerata* and *Octopus bimaculoides*. [File_S2_NPP_summary.pdf]. File S3. Description of *Haliotis laevigata* neuropeptides identified during maturation. [File_S3_Maturation_mass_spectral_analysis.xlsx]. File S4. Neuropeptides detected during sexual maturation in *Haliotis laevigata*. SE – standard error; VGI – visual gonad index; NS-non-significant; *P* < 0.1; * *P* < 0.05; ** *P* < 0.01; *** *P* < 0.001; - not detected; a – detected in males only; eos – end of sequence; sp – signal peptide. [File_S4_Maturation_statistics.xlsx]. Supplemental material available at FigShare: https://doi.org/10.25387/g3.9414512.

## Results and Discussion

### Overview of the Haliotis laevigata draft genome

The greenlip abalone (*Haliotis laevigata*) genomic DNA was sequenced and assembled into a draft 1.76 Gb genome (expected 1.54 Gb) with 63,588 scaffolds (>2kb) and an N50 of 86,085 bp (using 140 Gb of paired-end or 80x coverage and 100 Gb mate-pair Illumina reads). This result is comparable to that reported for the 1.8 Gb draft genome of the Pacific abalone (*Haliotis discus hannai*) (Nam *et al.* 2017). Transcriptome data (71 Gb) from eleven different tissues were generated to assist with gene annotation and to putatively identify 55,164 genes, of which 54,512 have transcriptome support (Table 1). The genome was annotated in a two-step process, first using expressed sequence tags, transcriptome and proteome data from *H. laevigata* and closely related species, then using *ab-initio* gene prediction trained on the results of the first annotation. Gene ontology, enzymes and protein signature data are available in File S1. Approximately 55% of transcripts had BLAST matches with the molluscan species, *L. gigantea*, *Crassostrea gigas* and *Aplysia californica* contributing 90% of matches (Note: the *H. discus hannai* genome was not available at the time of annotation) (Figure S1). The top five biological processes identified were cellular, metabolic, single-organism, localization and biological regulation. Importantly, signaling, reproduction and reproductive processes were also identified (Figure S1). The top three molecular functions and cellular components were binding, catalytic activity and transporter activity, and membrane, cell and organelle components respectively (Figure S1). The annotated genome and the predicted protein resources generated was intended to further our understanding of the function of genes involved in maturation and spawning in abalone; therefore both quantitative and qualitative analyses were applied to assess genome quality and completeness. First, a quantitative assessment against a metazoan single copy ortholog evolutionary conserved gene dataset of 978 genes (BUSCO) (Simão *et al.* 2015) was used to estimate that the draft genome is 86.6% complete with a duplication level of 2.2%. An additional 8.8% of genes were fragmented, while 4.6% were missing (Table 1).

**Figure 1:**
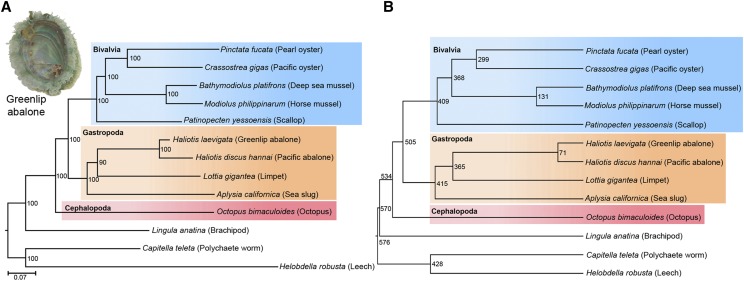
Phylogeny of *Haliotis laevigata* (greenlip abalone). (a) Maximum likelihood tree showing the phylogenetic relationship between ten molluscan species (one brachipod and two annelids were used as outgroups). This tree was based on 748 orthologous genes (127,598 amino acids) present in thirteen species. The robustness of the relationship was evaluated using the bootstrap re-sampling procedure, and the values based on 100 pseudo-replicates are given on bifurcating nodes. (b) A linearized time tree based on the *in-silico* proteomes of eleven Lophotrochozoan species and two annelids. The divergence times were estimated using a Bayesian Markov chain Monte Carlo method. The estimations were based on the maximum likelihood tree, which was calibrated using four well-defined fossil ages.

Second, qualitative assessment of the *H. laevigata* genome was conducted to compare the *in-silico* proteome of *H. laevigata* with other available complete molluscan and brachiopod *in silico* proteomes (https://www.ensembl.org/; https://www.ncbi.nlm.nih.gov/genbank/) (Takeuchi *et al.* 2012; Nam *et al.* 2017; Sun *et al.* 2017; Wang *et al.* 2017). This comparison was made using the maximum likelihood method (Stamatakis 2014), with annelids as outgroups (Figure 1). The inferred relationships between gastropods are in agreement with those reported in a recent study (Zapata *et al.* 2014). The observed relationships among molluscan species were similar to those reported by other genome-based studies (Sun *et al.* 2017; Wang *et al.* 2017). The rate of protein evolution appears to be relatively slower in greenlip abalone than in Pacific abalone (*H. discus hannai*) or the other two gastropods: *L. gigantea* and *A. californica*. The divergence time between the two abalone genomes was estimated to be 71 million years (MY) (highest posterior density 37-130 MY) (Figure 1). This is the youngest split-time estimate obtained and reported for molluscan species based on *in-silico* proteome data. In comparison, this divergence time is almost equal to that between humans and mice (dos Reis *et al.* 2012).

### Abalone neuropeptides

**Figure 2:**
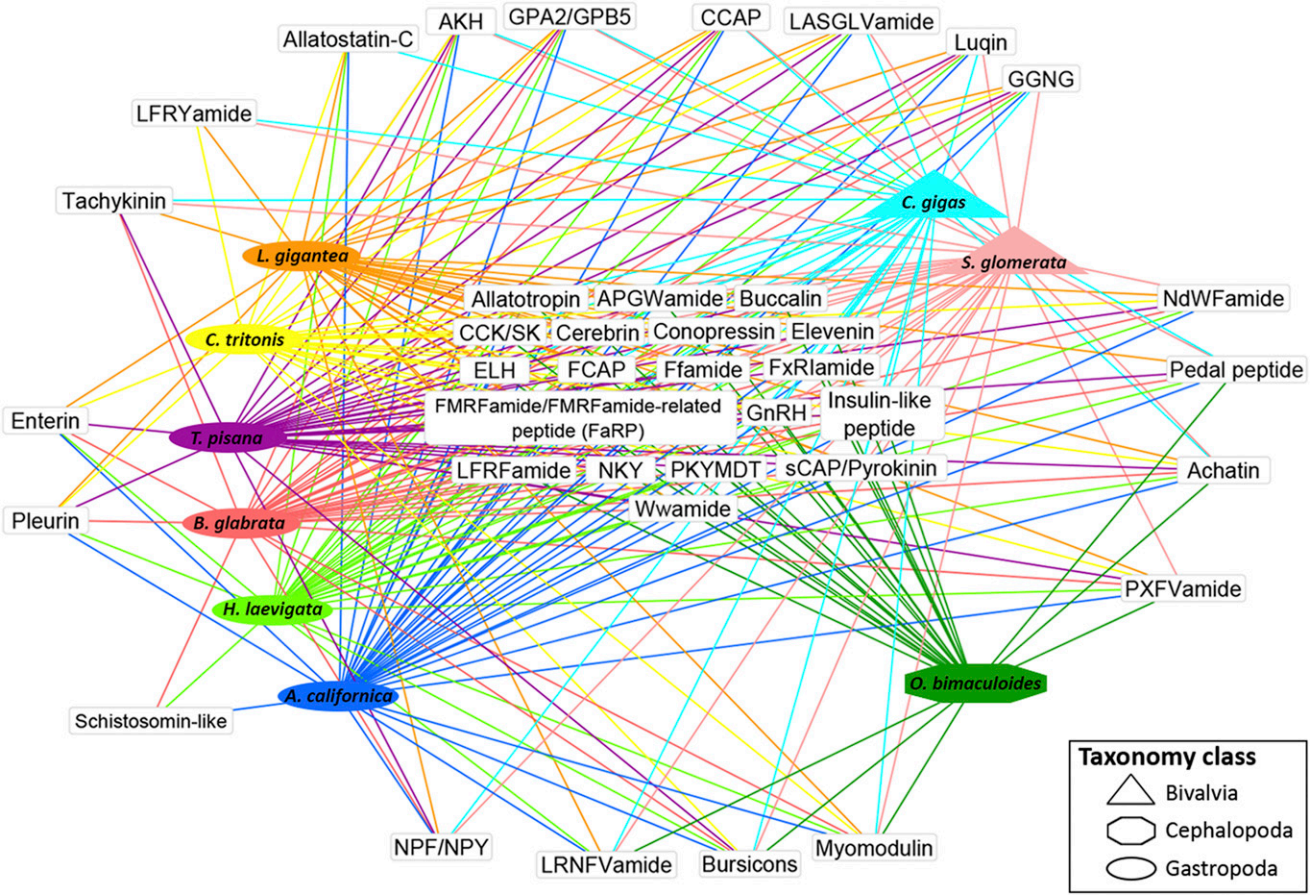
Neuropeptide precursor clustering across different molluscan classes. Included are: class Gastropoda (greenlip abalone *Haliotis laevigata*, Mediterranean white land snail *Theba pisana*, California sea slug *Aplysia californica*, owl limpet *Lottia gigantea*, Giant triton snail *Charonia tritonis*, marsh snail *Biomphalaria glabrata*), class Bivalvia (Pacific oyster *Crassostrea gigas*, Sydney rock oyster *Saccostrea glomerata*) and class Cephalopoda (California two-spot octopus *Octopus bimaculoides*). The total number of neuropeptides is 64 (for the list of NPPs and abbreviations, refer to File S2).

**Figure 3:**
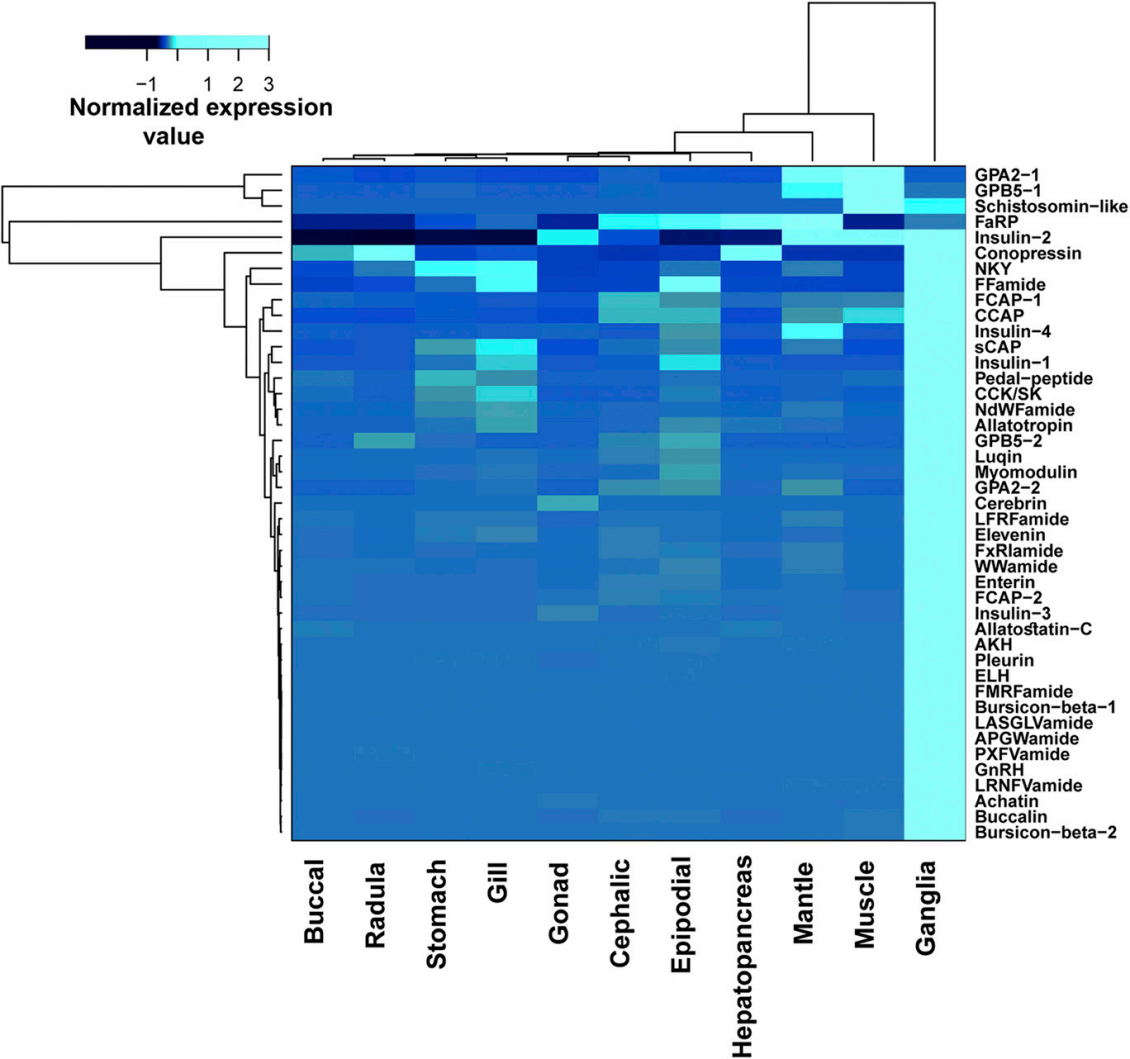
Tissue-specific expression of 43 neuropeptide precursor genes in *Haliotis laevigata*. Heatmap shows hierarchical clustering and normalized expression abundance across eleven tissues of neuropeptide precursor genes.

Controlled maturation and spawning of abalone is critical to the success of abalone aquaculture. Synchronous maturation of gonads is essential to production of quality seed and the ability to co-ordinate the spawning time of elite stock in the hatchery. Current methods to artificially induce spawning in abalone use either hydrogen peroxide or ultraviolet irradiation (or ozone) to generate free radicals in the water. This treatment is combined with the application of a temperature gradient to the water to induce spawning via a stress response (Kikuchi and Uki 1974). It is the animals neuroendocrine system, including secreted signaling neuropeptides, that help to integrate and regulate such behavioral states (Hahn 1992). Therefore, we used the draft genome to identify 45 neuropeptide precursor (NPP) genes (File S2). A comparison of NPPs present within the class Gastropoda (*H. laevigata*, *Theba pisana*, *A. californica*, *L. gigantea*, *Charonia tritonis*, *Biomphalaria glabrata*), class Bivalvia (*C. gigas*, *Saccostrea glomerata*) and class Cephalopoda (*O. bimaculoides*) demonstrates that 19 NPPs are conserved, including the reproductive-associated neuropeptides ELH, buccalin and gonadotropin-releasing hormone (GnRH) (Figure 2). The schistosomin-like, pleurin and enterin neuropeptides appear to have a more defined presence within the molluscan classes, specific to the gastropods. The schistosomin-like neuropeptide gene is known to be upregulated within fast growing tropical abalone (*Haliotis asinina*) (York *et al.* 2012b). Excluding the *H. laevigata* ganglia, 15 NPP genes show relatively high normalized expression in several other tissues, including the buccal, hepatopancreas, gill, stomach, radula, sensory tentacles (cephalic and epipodial), and foot muscle tissues (Figure 3). In contrast, gonad tissues exhibited detectable expression of only the conopressin, insulin-2 and cerebrin NPP genes.

### Reproductive maturation

*H. laevigata* become sexually mature at about 3 years of age (Shepherd and Laws 1974), however, their size (weight and length) are also determinants of reproductive maturation. (Shepherd and Triantafillos 1997; McAvaney *et al.* 2004; Wells and Mulvay 2005). This plasticity in maturation with age has also been shown for the New Zealand temperate abalone *Haliotis iris*, as well as other marine invertebrates (Thompson 1979; McShane and Naylor 1995; Franz 1996). The abalone used for this reproductive maturation study were of different ages, entering their 1^st^ and 3^rd^ years of maturation but of similar size throughout, attaining a length of 71.2 cm and 65.3 cm, respectively, at the peak of this species spawning season (December). The difference in size can be attributed to harvesting of the larger abalone in the commercial population prior to collection of these samples. McAvaney *et al.* (2004) hypothesized that size combined with associated physiological characteristics may be a limiting factor in inducing individuals to spawn. Toward exploring this, the relative abundance of neuropeptides in neural tissue may be a good indicator of the likelihood of successful spawning, particularly in younger abalone.

**Figure 4:**
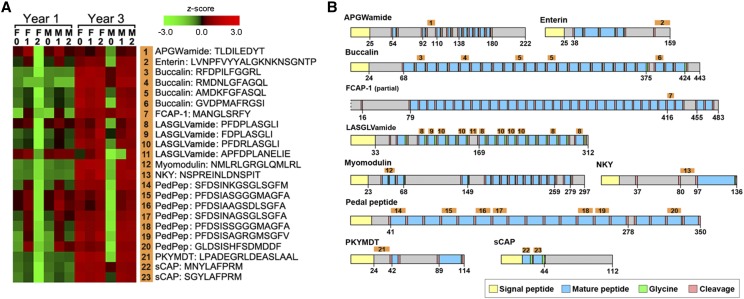
Neuropeptide identification in *Haliotis laevigata* central nervous system. (a) Neuropeptides detected and normalized abundance of each peptide in the central nervous system, in years 1 and 3 of reproductive maturation. The stages of gonad maturation are represented by the visual gonad indices (0-2). F, represents female and M, represents male. (b) Schematics of neuropeptide precursors showing the position of mass spectrometry peptides.

Using the *H. laevigata* NPP database, we conducted a mass spectral analysis (File S3) on the CNS of abalone (both males and females) entering the 1^st^ and 3^rd^ years of reproductive maturation, where gonad maturation was scored based on a visual gonad index (VGI) of 0 to 3 (Kikuchi and Uki 1974). This resulted in 23 peptides (derived from 10 NPPs) identified as showing significant changes in abundance (*P* < 0.05) between both reproductive stages (Figure 4; see File S4). It is noteworthy, that not all peptides matched NPP regions with known bioactivity, such as the TLDILEDYT and LVNPFVYYALGKNKNSGNTP peptides, which are derived from APGWamide and enterin-1 NPPs, respectively (Figure 4). In the 3^rd^ year of female reproductive maturation, the majority of peptides are consistently abundant in gonads with a VGI of 0 to 2, while in males there is a significant increase (*P* < 0.05) in gonads with a VGI of 0 to 1 (Figure 4). In the 1^st^ year of female and male reproductive maturation, only specific pedal peptides show consistent elevated abundance, and all peptides were largely absent in females with a VGI of 2. This is consistent with the transition to sexual maturity occurring at a size of between 65 and 95 mm in length (Officer 1999; Tarbath 2006) when the physiological abundance of neuropeptides has yet to peak as shown in abalone entering their third year of reproduction (Figure 4). Complete sexual maturation predominately occurs at three years of age or 75 to 120 mm for *H. laevigata* (Shepherd and Laws 1974) and may be a contributing factor in the limited success of spawning young, fast growing *H. laevigata* up to one year ahead of normal sexual maturity (McAvaney *et al.* 2004).

Some of the differentially abundant neuropeptides identified here have been implicated in abalone reproduction, or in other molluscs. For example, the APGWamide neuropeptide can stimulate maturation and spawning in the abalone *H. asinina* and *H. rubra*, as well as the Sydney Rock Oyster *S. glomerata*, (Chansela *et al.* 2008; York *et al.* 2012a; In *et al.* 2016). APGWamide associated with spawned oyster sperm has also been suggested to act as a pheromone to trigger female spawning (Bernay *et al.* 2006), which is consistent with some aquaculture practices of adding sperm to female spawning tanks. Another neuropeptide with evidence for reproductive regulation in molluscs is myomodulin, which is upregulated during maturation and spawning in *H. asinina* (York *et al.* 2012a). For other neuropeptides identified, there is evidence for regulatory roles in molluscan feeding (enterin-1, buccalin, and NKY) (Furukawa *et al.* 2001), muscle contraction (*e.g.*, pedal peptides, myomodulin) (Hall and Lloyd 1990) and gonad maturation (buccalin) (In *et al.* 2016).

### Spawning

**Figure 5:**
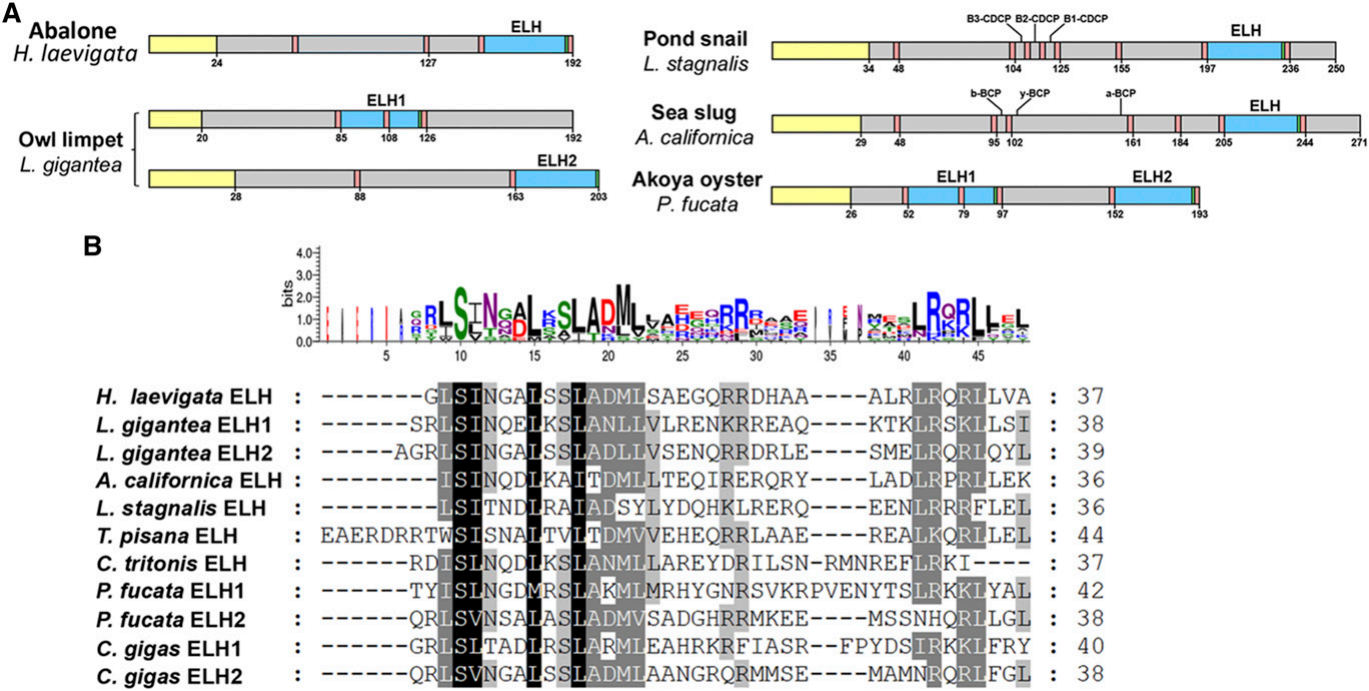
Identification and comparative analysis of egg laying hormone (ELH). (a) Schematics showing ELH precursor organization. BCP, bag cell peptide; CDCP, caudodorsal cell peptide. (b) Multiple sequence alignment of molluscan ELH. Shading in alignment shows levels of conservation. Sequence logo (above alignment) also provides an indication of amino acid conservation.

A number of neuropeptides that are well known for their role in animal reproduction were not identified in our CNS reproductive maturation proteomic investigation, include the ELH (Cummins *et al.* 2010; Stewart *et al.* 2016). This may be explained by insufficient quantities for detection until the final mature or ripe stage of gonad development (*i.e.*, VGI 3) in preparation for spawning. Or, ELH may be better attributed to stress-induced spawning. The *H. laevigata* genome-encoded ELH neuropeptide shows conservation with ELHs from other species, specifically within their bioactive peptide regions (Figure 5). The *H. laevigata* ELH NPP contains one copy of ELH, similar to that found in other gastropods. To investigate its potential function in spawning induction we performed intramuscular injection of synthetic ELH (1.0 µg/g body weight) into 3-year-old abalone broodstock conditioned to a VGI of 2. We found that a single injection of ELH was capable of inducing spawning in 80% of individuals at 7–20 h post-injection (Table 3). This suggests that a spike in ELH is required to induce spawning. An equal number of individuals were successfully spawned for both sexes suggesting that ELH is not sex-specific in *H. laevigata*. However, we acknowledge that increasing the number of biological replicates will be helpful to further verify results.

**Table 3:** Synthetic *Haliotis laevigata* egg laying hormone (ELH) functional assay results

Group	Mean weight (g)	Spawn	Unconfirmed spawn	Failed spawn	Observed pre-trail VGI *^a^*	Observed post-trial VGI
Control	82.3	0	0	5	2.0	2.0
ELH	77.0	7	1	2	2.0	0.5 (n = 8)
						2.0 (n = 2)

a Visual Gonad Index.

We have assembled a complete first draft *H. laevigata* genome assembly, which has been verified as useful through phylogenetic analyses, experimental and functional bioassays. We investigated the genomic architecture of this draft, with particular reference to genes likely to play a role in abalone reproduction. Importantly, we elucidated several neuropeptides with functional properties in abalone ELH one of which stimulated spawning in 80% of conditioned abalone. This research helps to position abalone in the modern era of genomics and will assist researchers to tackle major threats to wild fisheries (*e.g.*, rapid response to disease outbreaks using genomic technologies) and increasing commercial aquaculture production. These data can be used in modern marine biotechnology, providing a foundation for the broad range of ongoing and future research projects aimed at identifying molecular markers (*e.g.*, genome wide association studies, heritable immune factors, population diversity) and, understanding the genetics of stress responses (*e.g.*, husbandry, disease management, environmental monitoring), chemosensory biology (*e.g.*, maximize fertilization success, feeding), nutrigenomics (*e.g.*, feed response, maximize productivity), and comparative genomics (*e.g.*, adaptation traits). This will enable the scientific community and industries to respond to challenges in sustainability and economic prosperity.
